# Adapted Screening Methods for Gestational Diabetes in Low‐Resource Settings—A Narrative Review of Observational Studies

**DOI:** 10.1155/jp/6473503

**Published:** 2026-10-30

**Authors:** Magdalena Kittelmann, Cécile Monod, Beatrice Mosimann

**Affiliations:** ^1^ Department of Gynecology and Obstetrics University Hospital Basel Basel Switzerland unispital-basel.ch; ^2^ Swiss Tropical and Public Health Institute Allschwil Switzerland swisstph.ch; ^3^ University of Basel Basel Switzerland unibas.ch

**Keywords:** gestational diabetes, low-resource settings, oral glucose tolerance test, screening

## Abstract

**Introduction:**

Gestational diabetes mellitus (GDM) affects approximately 14%–17% of pregnancies worldwide, with the vast majority of cases occurring in low‐resource settings. There is an ongoing debate concerning the optimal screening and diagnosis of GDM, as the current diagnostic recommendation of performing a universal oral glucose tolerance test (OGTT) for every pregnant woman (WHO, 2013) is resource‐intensive and can potentially lead to overdiagnosis. This narrative literature reviews aimed to evaluate the diagnostic sensitivity of alternative, less resource‐demanding screening methods that could be appropriately used for low‐resource settings.

**Methods:**

For this narrative review, observational studies were identified via PubMed. The PRISMA guidelines were used to guide this process. As the studies investigated very diverse screening methods, no statistical meta‐analysis was performed due to methodological heterogeneity; instead, the results were synthesized narratively.

**Results:**

Ten studies were included, examining various alternatives such as fasting plasma glucose thresholds (sensitivity 95.6%), selective screening strategies (sensitivity 73.6%), capillary blood glucose testing (sensitivity 67.9%, 79.3%, and 92.5% for different fasting cut points), and glycated hemoglobin (HbA1c) measurements (sensitivity approximately 84%, depending on the cut‐offs).

**Discussion:**

All alternatives showed inferior sensitivity compared to the standard OGTT; therefore, none can currently substitute the OGTT. However, basically all approaches have the potential to either reduce the number of OGTTs or improve access to GDM screening for more patients. Further research and validation are necessary for all approaches to safely reduce the number of OGTTs without compromising diagnostic accuracy.

## 1. Introduction

Worldwide, gestational diabetes mellitus (GDM) affects approximately 14%–17% of pregnant women [1, 2]. According to the 2025 International Diabetes Federation (IDF) Diabetes Atlas an estimated 19.7% of women giving birth are affected by hyperglycemia in pregnancy, with GDM accounting for 79.2% of cases [3]. However, accurately estimating the prevalence of GDM remains challenging, as it is influenced by population‐specific risk factors, ethnicity and different screening strategies [4]. Most likely over 90% of patients with GDM live in low‐income and middle‐income countries (LMICs) [5, 6]. Because of lifestyle changes, these numbers are expected to rise even further in the coming years [7].

GDM is a condition that is first diagnosed during pregnancy [8]. The reasons are hormonal changes in pregnancy, leading to insulin resistance [4, 9]. Maternal and fetal complications can result from insufficient treatment of GDM, with possible consequences both in the short and long term [4]. Despite GDM being one of the most common conditions affecting pregnancy, there is no uniformity in the guidelines concerning screening methods [10]. The recommendations differ in terms of “universal versus selective screening, the optimal time for screening, appropriate tests and cut‐off values, and whether testing should be conducted in one or two steps” [11].

In 2013 the World Health Organization (WHO) adopted the IADPSG consensus criteria, which are based on the results of the multinational HAPO study [4, 8]. The HAPO study enrolled a study population of 25,505 pregnant women from 15 study centers in nine different countries; however, no study center was located on the African continent [8]. Because the study centers were mainly located in high‐income countries, the specified thresholds, especially for settings in Sub‐Saharan Africa need further evaluation [8, 12]. Consequently, the extent to which the IADPSG criteria can be appropriately applied to low‐resource settings remains unclear. The IADPSG recommends a universal one‐step screening in the form of a 75 g oral glucose tolerance test (OGTT) conducted between 24 and 28 weeks of gestation. Patients are required to drink 75 grams of glucose after an overnight fasting period for at least 8 h. GDM is diagnosed if one or more of the following values are elevated: “fasting plasma glucose (FPG) ≥ 5.1 mmol/L (92 mg/dL), 1 h ≥ 10 mmol/L (180 mg/dL), and 2 h ≥ 8.5 mmol/L (153 mg/dL)” [11]. The timeframe (24–28 weeks of gestation) is chosen due to the fact, that maternal blood glucose levels are physiologically lower during early pregnancy [11].

The WHO 2013 criteria are stricter than the previously recommended WHO 1999 criteria [12]. This implies that adopting the WHO 2013 criteria results in a 2.5‐ to 3‐fold increase in the number of patients diagnosed with GDM. This may place an additional burden on health systems potentially leading to overdiagnosis, as differences in the maternal and neonatal outcomes are questionable [13–15].

Screening using the one‐step fasting OGTT requires a lot of resources, which are often unavailable in low‐resource settings [9]. The test should be performed between 24 and 28 weeks of gestation and requires repeated venous blood sampling [16, 17]. In addition, the OGTT is often poorly accepted by pregnant women because of the required efforts [18]. Patients must fast beforehand; the procedure is time‐consuming and the ingested glucose often causes nausea [17, 19]. Consequently, screening uptake varies widely in LMICs, from 10% to over 90% [6], in certain settings, many pregnant women are not screened at all [5, 20].

The WHO strongly supports research on effective screening methods of GDM in low‐resource settings [21, 22]. Besides the burdensome OGTT also other methods are used, such as the measurement of random blood sugar (RBS), fasting blood sugar (FBS), and glycated hemoglobin (HbA1c) [23], even if they lack scientific evidence. However, some clinical observational studies have already been conducted in low‐resource settings to evaluate the accuracy of these alternative screening methods (see Table 1).

**Table 1:** Study synopsis of the narrative review showing the study characteristics.

Nr.	Authors	Year	Title	Country	Study design	Study period	Sample size	Screening method and diagnostic criteria
1	Agarwal [24]	2018	“Gestational Diabetes Mellitus: Using the Fasting Plasma Glucose (FPG) Level to Simplify the IADPSG Diagnostic Algorithm in an Adult South Asian Population”	India	Cross‐sectional study	January 2013–December 2015	6.520	OGTT (WHO 2013) was performed at 24–28 weeks of gestation, FPG performance for diagnosis of GDM was assessed
2	Khambule [25]	2026	“Fasting Plasma Glucose Performs Better Than Glycated Hemoglobin and Glycated Albumin for Diagnosing Gestational Diabetes”	South Africa	Cross‐sectional study	November 2018–May 2022	524	OGTT (WHO 2013) was performed between 24 and 32 weeks of gestation in the presence of risk factors, versus HbA1c, glycated albumin (GA) and FPG.
3	Nwali [11]	2021	“Universal versus Selective screening for GDM Among Antenatal Clinic Attendees in Abakaliki: Using the One‐Step 75 Gram OGTT”	Nigeria	Cross‐sectional comparative study	November 2017–April 2018	400	OGTT (WHO 2013) at 24–28 weeks of gestation versus risk factor screening for GDM
4	Olagbuji [12]	2015	“Prevalence of and Risk Factors for GDM Using 1999, 2013 WHO and IADPSG Criteria Upon Implementation of a Universal One‐Step Screening and Diagnostic Strategy in a Sub‐Saharan African Population”	Nigeria	Prospective observational study	November 2012 and March 2014	1.059	OGTT (WHO 2013) at 24–32 weeks of gestation in a universal one‐step screening versus selective screening
5	Bhavad‐harini [26]	2016	“Use of CBG for Screening of GDM in Low‐Resource Settings”	India	Cross‐sectional study	Study is part of the program Women in India with GDM Strategy (WINGS)	1.031	OGTT (WHO 2013) with VPG and simultaneously CBG
6	Putoto [16]	2020	“A Simplified Diagnostic Work‐Up for the Detection of Gestational Diabetes Mellitus in Low Resources Settings: Achievements and Challenges”	Sierra Leone	Cross‐sectional study	March 2018–January 2019	5.799	OGTT was prescribed after 24 weeks′ gestation to women with risk factors and borderline glucose values at baseline evaluation. Blood sugar testing was done with CBG. Primary aim: determining the rate of women that could be assessed with this selective screening policy.
7	Punnose [17]	2020	“HbA1c in the First Trimester: A Predictor of Gestational Diabetes Mellitus in Pregnant Asian Indian Women”	India	Retrospective cohort study	January 2011 and December 2016	2.275	HbA1c first trimester estimation versus GDM screening with a one‐step OGTT (WHO 2013)
8	Saravanan [5]	2024	Early Pregnancy Hba1c as the first Screening Test for Gestational Diabetes: Results From Three Prospective Cohorts	India, Kenya, United Kingdom (multiethnic cohort)	Prospective cohort stud	February 2016, December 2019	STRiDE cohort: 3.070 India, 4.104 Kenya PRIDE cohort: United Kingdom 4320	Early pregnancy HbA1c versus OGTT (WHO 2013) at 24–28 weeks of gestation
9	Dubey [18]	2019	“Mid‐Trimester HbA1c and its Correlation With OGTT (World Health Organization 1999)”	India	Prospective observational study	Period of 1 year	260 (220 performed OGTT)	OGTT 24‐28 week of gestation (WHO 1999‐criteria) versus simultaneous HbA1c
10	Soumya [27]	2015	“HbA1c: A Useful Screening Test for GDM”	India	Prospective observational study	Not named	500	OGTT (WHO 2013) and HbA1c were performed and compared between 24 and 28 weeks of gestation and in women with GDM at 6 weeks postpartum

The objective of this narrative literature review is to evaluate whether more resource‐saving and therefore cost‐effective screening methods with high sensitivity can be recommended for low‐resource settings. Such methods could potentially still ensure diagnostic accuracy as they could still diagnose most pregnant women with GDM.

## 2. Methods

The study was conducted as a narrative review. Although the review is oriented toward the PRISMA guideline for systematic reviews [28], the study selection and data extraction were performed by a single author. The search strategy follows the PICO/PECO structure, a framework for developing clinical questions. The acronym stands for population, intervention/exposure, comparison and outcome. Adapted to this narrative review, the study population comprised pregnant women in low‐resource settings who underwent screening for GDM (the intervention/exposure). The standard diagnostic test, a one‐step universal screening with 75 g OGTT recommended by the WHO since 2013, served as the reference comparison [29]. Studies addressing the sensitivity, safety, and cost‐effectiveness of GDM screening during pregnancy in low‐resource settings were searched for.

The inclusion criteria were that the observational studies had to be conducted in a low‐resource setting and be published in English. The term “low‐resource setting” refers to studies conducted in low‐, lower‐middle‐, and upper‐middle‐income countries according to the World Bank classification. The timeframe for the selected studies was set as 2013–2026. Studies were excluded if they addressed diabetes mellitus type I and II (instead of GDM), included twin pregnancies, had no full text available, did not match the PICO/PECO structure, or did not answer the research question. Only original quantitative studies were included whereas qualitative studies and systematic reviews were considered solely for contextual background information but were not included in the study synopsis.

The search was conducted in PubMed on the 28.04.2025 using the following search strategy, containing key words and MESH‐terms:

((“Diabetes, Gestational”[Mesh]) OR (“Pregnancy”[Mesh])) AND ((LMICs) OR (Low and Middle Income Countries) OR Subsaharan Africa OR “Africa”[Mesh] OR “Asia”[Mesh])) AND ((“Diagnosis”[Mesh]) OR (“Diagnostic Techniques, Obstetrical and Gynecological”[Mesh]) OR (“Prenatal Diagnosis”[Mesh]) OR (“Risk Factors”[Mesh])) AND ((“glucose tolerance test”[MeSH Terms] OR OGTT [Text Word])) AND ((“Economics”[Mesh]) OR (“Pregnancy Complications”[Mesh]) OR (“Sensitivity and Specificity”[Mesh])) AND (2013:2025[pdat]).

The literature search was updated on May 25, 2026 during the review process. Newly identified records were screened using the same eligibility criteria and incorporated into the review. Following the initial search, titles and abstracts of all the studies identified were evaluated using the PICO/PECO structure. Systematic reviews and other studies touching on similar topics were also searched for contextual information. Furthermore, international guidelines and consensus statements including reports from the WHO and the International Federation of Gynecology and Obstetrics (FIGO), were also considered for background knowledge. However, a systematic search for gray literature was not conducted within the scope of this narrative review. Next, a full‐text screening was conducted, applying the eligibility criteria in addition. Following these steps, the relevant data were extracted from every selected article. A data extraction form example can be found in Table S1. Quality assessments were conducted using the Axis appraisal tool for cross‐sectional studies and the CASP appraisal tool for prospective and retrospective observational cohort studies (Tables S2 and S3). As the included studies were conducted on very different interventions, using different methods and outcomes, it was impossible to conduct a meta‐analysis or statistical correlation. Nevertheless, the characteristics of the studies and the interventions (i.e. the different screening methods evaluated in the studies), as well as the studies′ recommendations, were summarized in a table and interpreted in the following discussion.

## 3. Results

The initial search identified 414 studies, after the update 67 studies were added (total number 481 studies). After screening the titles and abstracts, 63 studies were included for the full‐text screening. Twelve studies met the eligibility criteria. However, two of these were subsequently excluded: one because the research question focused primarily on the prevalence of GDM rather than on screening methods, and another because it questioned the validity of the OGTT rather than finding valid alternatives [30, 31]. The remaining 10 studies were included in this narrative review (Figure 1). The studies excluded at the full‐text screening stage mainly evaluated risk‐based screening models that could potentially be applied as resource‐saving screening models in the future. However, none of these models are currently applicable; therefore, these studies were not chosen for the synopsis. Furthermore, studies with a design other than that of an observational study were also excluded.

**Figure 1:**
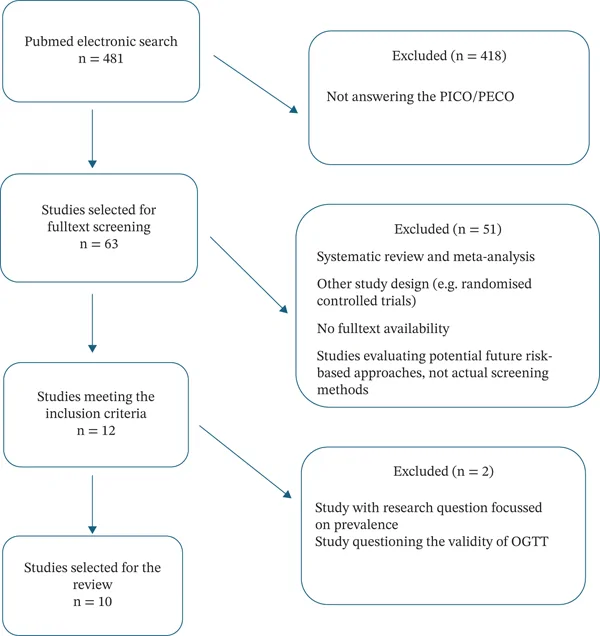
Flow diagram for the study selection process.

The 10 included studies consisted of five cross‐sectional studies, four prospective cohort studies, and one retrospective cohort study. The publication dates of these articles ranged from 2015 to 2026. Most of the studies originated in India, although one study was conducted in a multiethnic cohort including Kenya and the United Kingdom, two studies originated in Nigeria, one study was conducted in Sierra Leone and one in South Africa. The sample size ranged from 220 to 7.174 participants. The study characteristic and the main results are depicted in Table 1 and Table 2.

**Table 2:** Study synopsis of the narrative review showing the results.

Nr.	Authors	Year	Method of analysis	Sensitivity (%), specificity (%)	Outcome
1	Agarwal [24]	2018	Area under the receiver operating characteristic curve was employed to examine the overall performance of the FPG to predict GDM. The sensitivity, specificity, positive and negative predictive values for differing threshold values of FPG and the likelihood ratios of positive and negative tests were calculated	Sens. 95.6, if FPG‐thresholds < 4.3 mmol/L and ≥ 5.1 mmol/L are used	Using two FPG‐thresholds (< 4.3 mmol/L and ≥ 5.1 mmol/L), only 52.5% women would need to proceed to the OGTT to detect 95.6% of women with GDM
2	Khambule [25]	2026	The area under the ROC curve (AUC) with 95% confidence interval (CI) were determined for GDM diagnosis using HbA1c, GA and FPG as continuous variables and the OGTT as the reference method. The optimum GA and HbA1c thresholds for diagnosing GDM were estimated using the Youden index. Sensitivity (%), specificity (%), positive predictive value (PPV) and negative predictive value (NPV) were calculated for each test at the optimum threshold.	HbA1c at a 5.5% cut‐point: sens. 54, spec. 80 GA at a 37.56% cut‐point: sens. 54, spec. 83. FPG cut‐point 5.1 mmol/L (92 mg/dL): sens. 87 and spec. 100, FPG cut‐point 4.6 mmol/L (83 mg/dL): sens. 90, spec. 75	FPG used in a two‐tiered process for identifying GDM cases can reduce the number of required OGTTs by 77% while maintaining high sensitivity and specificity. HbA1c and GA are not appropriate diagnostic markers for GDM.
3	Nwali [11]	2021	Statistical comparison was done using *t*‐test for continuous variables. Logistics regression was used to determine the level of associations of the independent predictors for hyperglycemia.	Sens. 73.58, spec. 48.82	Universal screening is superior to selective risk factor‐based screening for GDM as it was able to identify 31.11% of patients missed on risk factor‐based screening. The predictors of GDM in this study are increased maternal age, obesity, history of previous GDM, and hypertension.
4	Olagbuji [12]	2015	Univariate logistic regression analysis was conducted to examine the unadjusted odds of gestational diabetes for each maternal clinical covariate in relation to a particular referent category. Clinical covariates with *p* < 0.05 on univariate analysis were tested using multivariate logistic regression to evaluate the adjusted odds.	Sens: 80.2, if WHO 2013 criteria are used. (Sens. calculated as 1 − proportion of GDM cases missed by selective screening)	Approximately 20% of GDM cases would have been missed using a selective screening strategy versus OGTT (new consensus diagnostic criteria)
5	Bhavadharini [26]	2016	Sensitivity, specificity, positive and negative predictive value, and accuracy of different CBG cut points were calculated for the IADPSG criteria. Bland–Altman plots were drawn to look at the agreement between CBG and VPG in fasting, 1H and 2H samples and to see whether it was within the 95% limits. Pearson′s correlation was used to compare CBG and VPG values at fasting, 1H and 2H time points.	Fasting CBG cut point 80 mg/dL (4.4 mmol/L): optimum sens. 67.9 and spec. 58.5. Cut point 75 mg/dL: sens. 79.3, spec. 36.9. Cut point 70 mg/dL: sens. 92.5, spec. 16.1. 2‐h CBG cut point 140 mg/dL (7.8 mmol/L), sens. 62.3, spec. 80.7. Cut point 120 mg/dL, sens. 78.3, spec. 53.1. Cut point 110 mg/dL, sens. 92.5, spec. 33.7.	In resource‐constrained situations where obtaining venous samples is impossible, the initial screening can be done by CBG using handheld glucose meters with lower 2H glucose cut points
6	Putoto [16]	2020	The 95% confidence intervals of proportions were calculated using a binomial distribution model. The Kaplan–Meier method was used to obtain the survival curve on the cumulative risk of GDM with gestational age. The frequency of risk factors between affected and unaffected women was compared using the Fisher′s exact test.		Three quarters of subjects could be assessed with our approach. The approach was only indirectly compared with OGTT, as the data were overall compared with previously reported prevalence, which are mostly based on OGTT
7	Punnose [17]	2020	Receiver operating characteristic (ROC) curve statistics were applied to assess the discriminative ability of HbA1c‐FT in GDM diagnosis. A multivariable logistic regression analysis after adjusting for plausible confounders was used to evaluate the independent effect of HbA1c‐FT on GDM diagnosis.	HbA1c–FT value of 5.6% (38 mmol/L), sens. 5.5, spec. 98.8. HbA1c–FT value 5.2% (33 mmol/L), sens. 30.7, spec. 84.2	First trimester HbA1c is an independent GDM predictor in Asian Indian women but lacks sufficient sensitivity or specificity for use as a diagnostic test
8	Saravanan [5]	2024	Logistic regression: Poisson regression models, adjusted risk ratio. ROC curves were used to identify the single best composite risk factor threshold for prediction of gestational diabetes	India: sens. 80.6 (HbA1c thresholds 4.9% and 5.4%) Kenya: sens. 80.0 (HbA1c thresholds 5.2% and 6.0%) United Kingdom: sens. 79.5 (HbA1c thresholds 5.2% and 5.6%)	Early pregnancy HbA1c was independently associated with incidence of GDM at 24–28 weeks of gestation. A population‐specific, two‐threshold screening strategy of rule‐in and rule‐out GDM using early pregnancy composite risk score could reduce the requirement of OGTTs by 50%–64%. Therefore, early pregnancy HbA1c offers a simple screening test for GDM, allowing those at highest risk to receive early intervention
9	Dubey [18]	2019	Mean and standard deviation for normal distributed variables and as median (range) for non‐Gaussian variables. Student′s *t*‐tests and other appropriate tests were used for testing significance. Receiver operating characteristic (ROC) curve was applied to analyze the performance of HbA1c test.	With WHO 1999 OGTT criteria, for HbA1c at a cut‐off ≥ 5.45% the sens. 84.3. spec. 81.8	HbA1c cannot replace the OGTT for screening but: HbA1c can be used as a single‐shot screening assessment for the diagnosis of GDM in order to avoid inconvenience: Despite being a costly test its advantages give it an upper edge to be used as simple and acceptable approach for screening of GDM.
10	Soumya [27]	2015	Multivariate linear regression was used to analyze the effect on continuous variable pregnancy outcomes with variables as gravida, parity, obstetric history, conception with assisted reproductive techniques, and hypothyroidism as covariates. Similarly, multivariate logistic regression was used to analyze categorical pregnancy outcomes. The receiver operator characteristic (ROC) curve was constructed using OGTT as the gold standard and HbA1c as the variable of interest.	HbA1c cutoff value 5.3%: sens. 95.6, spec. 51.6. HbA1c cutoff value 5.7%: sens. 73.3, spec. 75.6. HbA1c cutoff value 6.1%, sens. 46.7, spec. 95.	Although HbA1c cannot replace OGTT in the diagnosis of GDM, it can be used as a screening test, avoiding OGTT in approximately 50% of women, if a cutoff of 5.3% is used

The studies included in the synopsis evaluate various screening approaches primarily in comparison to the WHO 2013/IADPSG consensus criteria. Two studies do not directly meet this criterion: Dubey et al. (2019) used the WHO 1999‐criteria as comparison [18], and Putoto et al. (2020) compared the results to reviews that are mostly based on the consensus criteria [16].

## 4. Discussion

In this analysis 10 studies were identified that evaluated alternative screening methods for GDM to the 75 g OGTT in LMICs. Overall, four different screening approaches are compared with the OGTT: screening by FPG, selective screening instead of universal screening, screening by capillary blood glucose (CBG) instead of venous plasma glucose and the assessment of HbA1c both in the first and second trimester. All included studies share the aim of avoiding costly and inconvenient OGTTs, which therefore miss out many patients who require GDM screening. Because of the very different study designs, it was not feasible to perform a meta‐analysis.

Previous studies have already evaluated the use of FPG and have shown a lower sensitivity for FPG compared with OGTT [27]. In the HAPO study, only around 52% of women with GDM were identified by FPG alone [27], the results differed widely for various settings from 24.0% in Thailand to 73.0% in the United States [24]. However, other studies have shown good sensitivity, depending on the chosen cutoff values [27]. For example, the national guideline in Switzerland recommends the use of FPG as rule‐in, respectively, rule out‐criterion for low‐risk patients, with sensitivity ranging from 78.5 to 95% [32, 33].

Also, the studies included in this review, showed positive results for the use of FPG. Soumya et al. (2015) evaluated FPG in addition to focusing on HbA1c values, and stated, that “a large majority (96%) of the women diagnosed with GDM had an abnormal FPG” [27]. Agarwal et al. (2018) evaluated FPG thresholds to avoid OGTTs [24]. The study found that “below 4.3 mmol/L and 5.1 mmol/L or above, only 52.5% women would need to proceed to the OGTT to detect 95.6% of women with GDM” [24]. Also, Khambule et al. (2026) showed that FPG cut‐points of 4.6 mmol/L (83 mg/dL) and 5.1 mmol/L (92 mg/dL) would reduce the number of OGTTs by 77% with a sensitivity of 90.0% and a specificity of 74.9% [25]. Agarwal et al. (2018) as well as Khambule et al. (2026) concluded that FPG screening represents a promising option, if certain preconditions are met. The FPG rule‐in and rule‐out thresholds must be evaluated for individual ethnic groups to maintain acceptable sensitivity levels [24, 25]. Although FPG screening cannot fully replace the OGTT, especially using a two‐threshold strategy can substantially reduce the number of required OGTTs while maintaining high sensitivity [24].

Besides using FPG for the GDM diagnostics, selective screening strategies based on clinical risk factors also need further evaluation. Few countries worldwide follow the NICE guideline that recommends a selective screening approach, whereas most guidelines (e.g. ACOG/FIGO) suggest a universal screening [11]. Selective screening strategies utilize risk factors that have been shown to increase the likelihood of developing GDM. These risk factors include among others the premedical history of GDM or macrosomic baby as well as intrauterine fetal death [11]. Other maternal risk factors include obesity, high blood pressure, smoking or higher age, and parity [11]. However, the available evidence indicates, that a significant proportion of patients with GDM are missed [11, 12].

The findings of the present review confirmed these observations. Nwali et al. (2021) tested a selective versus universal screening approach [11]. The participants in the selective group were chosen based on having “one or more risk factors for GDM” [11]. A multivariate analysis revealed a significant association with the development of hyperglycemia only for the following variables: age over 35, weight during pregnancy over 90 kg, GDM in a previous pregnancy, and known hypertension [11]. Olagbuji et al. (2015) also evaluated a selective screening approach at a point of time when universal screening was introduced at the hospital that served as the study location [12]. Before universal screening was introduced, selective screening was performed on patients with at least one risk factor in accordance with the National Institute for Health and Care Excellence (NICE) guidelines [34]. Both Nwali et al. (2021) and Olagbuji et al. (2015) concluded that the numbers of patients being missed in the selective screening (20%–30%) was too high to recommend this approach [11, 12].

Another practical issue in low‐resource settings is addressed by evaluating the clinical utility of CBG. This is also a valuable approach, as laboratory facilities for estimating venous plasma glucose are not available in some low‐resource settings [26]. Furthermore, using CBG is an obvious idea, as treatment blood glucose levels are tracked by using CBG in patients with GDM. However, the blood glucose levels vary between capillary and venous blood sampling. To compensate for this variation, WHO recommendations for adapted cutoff values currently only exist for nonpregnant adults [26].

In this review, Bhavadharini et al. (2016) found that an OGTT could be conducted cost‐effectively as an initial screening by using capillary blood with adapted thresholds for blood sugar measurements instead of venous blood [26]. The results show that this method could only be used as a screening tool if lower cutoff points are applied; a referral to a higher‐level healthcare facility would be necessary for the final diagnosis of GDM [26]. Putoto et al. (2020) attempted to develop a suitable screening strategy for low‐resource settings, combining a selective screening approach with CBG [16]. However, no adapted thresholds for the CBG measurement were employed [16]. A screening at any gestational age was combined with an OGTT after 24 weeks of gestation, following a risk factor assessment, respectively, when GDM was suspected at the baseline evaluation. According to the results of the study, this approach resulted in three‐quarters of the cases being detected [16]. However, this approach still requires further evaluation in different settings.

The use of HbA1c was evaluated during the first and second trimesters. The advantage of measuring HbA1c is that only a single venous puncture is required; neither fasting nor ingesting a glucose solution is necessary [18]. In 2011, California introduced a program that did not use an OGTT for confirmation, if HbA1c values were between 5.7 and 6.4% in early pregnancy [17]. Since some settings already follow the IADPSG recommendation that HbA1c should be tested at the first antenatal visit to exclude overt diabetes, it is reasonable to use this test also for GDM diagnosis as well [17]. However, difficulties arise when using HbA1c because levels differ physiologically between the trimesters of pregnancy and values may be inaccurate in patients with anemia, which is a common condition in pregnant women, particularly in low‐resource settings [18].

The use of HbA1c testing was the focus of four studies in this review. Punnose et al. (2019) and Saravanan (2024) examined the results of the HbA1c testing during the first trimester as a marker for the development of GDM [5, 17]. Punnose et al. (2019) suggested that HbA1c‐FT could be used as an independent predictor of GDM, but not as a diagnostic test [17]. Saravasan et al. (2024) found different thresholds for different settings that could at least reduce the burden of the OGTT screening by more than 50%. However, the thresholds would need to be adapted for each setting; the rule‐in and rule‐out criteria ranging from 5.4% and 4.9% in India to 6.0% and 5.2% in Kenya [5]. Dubey et al. (2019) and Soumya et al. (2015) took a slightly different approach, comparing HbA1c values in weeks 24 to 28 of gestation with OGTT results taken at the same time points. Dubey et al. (2019) found that the HbA1c could not replace OGTT screening, with sensitivities, specificities, positive predictive values, negative predictive values, and accuracies of 84.3%, 81.8%, 53.1%, 95.5%, and 82.3%, respectively, if the HbA1c cut‐off was ≥ 5.45% [18]. However, the authors stated that HbA1c could potentially be used as a simplified approach [18]. Soumya et al. (2015) showed similar results; however, they argue that the OGTT could be avoided in about 50% of cases, by using an HbA1c cutoff of 5.3% [27].

Overall, the four studies included in this review that evaluated the use of HbA1c showed that it cannot replace the OGTT [5, 17, 18, 27]. Additionally, Khambule et al. (2026) have assessed HbA1c values alongside the use of glycated albumin (GA) and FPG, also confirming insufficient sensitivities (53.7% and 53.3%) for these screening parameters [25]. However, by specifying cutoff values for different ethnicities and combining rule‐in and rule‐out criteria, the numbers of OGTTs could potentially be reduced with this more patient‐friendly approach [5, 18, 27]. As conducting the HbA1c test is relatively expensive, economic considerations regarding cost‐effectiveness may also play a role in future research on this topic [18].

Cost analyses should also be conducted for many other aspects of GDM screening. On the one hand, consideration should be given to the different direct and indirect costs of GDM screening protocols. On the other hand, the question of what could create more costs has to be raised: whether greater expenditure arises from overdiagnosis and overtreatment or undetected GDM with subsequent short‐term and long‐term consequences for mother and child. Consequently, comprehensive cost‐effectiveness analyses of the different short‐term and long‐term financial burdens should be conducted in future. Overall, it is interesting to note that the WHO 2013 recommendation is being actively questioned, as it does not seem fully applicable to many low‐income settings worldwide. The various studies included in the present narrative review suggest different ways of screening for GDM, trying to circumvent the resource‐intensive one‐step OGTT. It has already been mentioned that the WHO 2013 recommendation, that is based on the HAPO study, remains controversial in resource‐constrained environments. Consequently, the extent to which the IADPSG criteria can be applied to low‐resource settings remains unclear. Additionally, some settings face other difficulties. A study conducted in Uganda showed that the 75 g OGTT, which was not evaluated in these circumstances, can lead to incorrect results. For example, food insecurity in the days before the test can confound maternal blood glucose values [31]. This demonstrates that gold standards for diagnosis and treatment often cannot be applied in low‐resource settings and that adaptation to local contexts may be necessary [16]. Additionally, a recent meta‐analysis comparing one‐step and two‐step screenings discovered no clear evidence that using a more sensitive screening method results in a better clinical outcome [35]. A study conducted in Japan also showed that pregnancy outcomes did not change when pregnant women with mild forms of GDM were treated [36]. Thus, adopting the IADPSG thresholds, that have the highest sensitivity, could potentially lead to overdiagnosis in certain settings [4]. Using the two‐step strategy could also be more acceptable and potentially more accurate in certain circumstances [5]. However, also another argumentation exists. According to the FIGO Update of 2021 a two‐step strategy could deteriorate the screening uptake in LMICs “because of multiple personal and health system factors, including scheduling issues, additional travel time, and cost” so moving back to a two‐step strategy is not recommended [6].

As a compromise, the DIPSI screening strategy is recommended in India. The advantage is that the DIPSI screening can be conducted in a non‐fasting state utilizing a single blood glucose value evaluated 2 h post‐glucose load. It is evident that this method offers advantages for low‐resource settings. However, a recent meta‐analysis reported a critically low sensitivity of 44% compared with the IADPSG criteria, so also this screening strategy is not advisable [37].

Instead, risk factor‐based models should be evaluated in further research, as they have the potential to save resources while hopefully maintaining an acceptable sensitivity [38]. Also, hybrid models for GDM screening need further development. For example, a recent study combined FPG and HbA1c thresholds; such combined approaches could work as a simplified screening method in low‐resource settings in future [39]. Also, other measures could be implemented to improve testing rates in a timely manner. For example, some studies have had success with the implementation of task shifting strategies: in a rural healthcare facility in Nepal, for instance, nurses carry out OGTTs [40]. A study in Nigeria revealed that health care workers have limited knowledge of hyperglycemia in pregnancy [41]. However, educational training is possible, and instructing community health workers in other settings could improve screening rates [10, 41].

Concerning GDM diagnostics and treatment, further aspects are to be discussed. A GDM diagnosis must be followed by immediate dietary recommendations or even oral antidiabetics or insulin treatment to avoid adverse pregnancy outcomes. In low‐resource settings, medication can be difficult to access and monitor, and dietary recommendations often do not align with local cultural or socioeconomic habits. For example, protein‐rich nutrition may not be available [10]. Consequently, when improving diagnostic algorithms, future research and clinical guidelines must consider these postdiagnostic therapeutic capacities.

The conducted narrative literature review has certain limitations. Only one database was searched in English language and only one author conducted the study selection process, which may have introduced a selection bias. Also, the fact that most included studies were conducted in India may limit the generalizability of the findings to LMICs in other geographic regions. The study review process was guided by the PRISMA framework; however, as this is a narrative review, the PRISMA protocol was not strictly followed. Potentially additional relevant studies may exist that were not captured within this scope.

Nevertheless, this review also has notable strengths. Applying the AXIS and CASP quality assessment guidelines revealed an acceptable overall quality percentage for the studies, ranging from 70%–80% for cross‐sectional studies and 75%–83% for prospective and retrospective observational cohort studies. Furthermore, this review provides a comprehensive synthesis of the most prevalent ideas on this important topic and identifies key knowledge gaps that may inform future research.

## 5. Conclusion

In summary, the study synopsis of this review demonstrates that various screening methods have been investigated to improve GDM diagnosis. Although there are some promising approaches, most proposed strategies still require further research and improvement. Besides further evaluating HbA1c measurements also implementing rule‐in and rule‐out FPG thresholds could help to circumvent nearly half of the OGTTs while maintaining high sensitivity [24]. Using CBG as a screening method in peripheral centers could also substantially enhance diagnostic accessibility for patients [16]. Further research could also identify valid and reproducible factors and biomarkers for selective risk‐stratified screening, which could at least diagnose high‐risk patients in settings where conducting a universal screening is not possible [16, 42]. Pilot screening programs should be implemented to validate alternative diagnostic approaches against the 75 g OGTT prior to their large‐scale adoption.

Ultimately, a single universal worldwide GDM screening strategy may be insufficient, and uniformity should not necessarily be the primary goal. Various screening strategies should be validated so they can be adapted to different settings while maintaining high sensitivity and demonstrating positive short‐term and long‐term effects for mothers and their offspring. Further research addressing the questions surrounding GDM in LMICs is highly encouraged, as GDM can have fatal consequences, especially in low‐resource settings where access to comprehensive emergency obstetric and newborn care may be limited [16].

**Nomenclature**
ACOG: American College of Obstetricians and Gynecologists
CBG: capillary blood glucose
FBS: fasting blood sugar
FIGO: International Federation of Gynecology and Obstetrics
FPG: fasting plasma glucose
GA: glycated albumin
GDM: gestational diabetes mellitus
HbA1c: glycated hemoglobin
HbA1c‐FT: first trimester glycated hemoglobin
IADPSG: International Association of Diabetes and Pregnancy Study Groups
IDF: International Diabetes Federation
LMICs: low‐income and middle‐income countries
NICE: National Institute for Health and Care Excellence
OGTT: oral glucose tolerance test
VPG: venous plasma glucose
PICO/PECO: population, intervention/exposure, comparison, and outcome
PRISMA: Preferred Reporting Items for Systematic Reviews and Meta‐Analyses
RBS: random blood sugar
ROC: receiver operating characteristic
sens: sensitivity
spec: specificity
WHO: World Health Organization

## Author Contributions

Magdalena Kittelmann designed the study and drafted the initial manuscript. All authors contributed to the critical revision of the manuscript. All authors conceptualized the work.

## Funding

No funding was received for this manuscript.

## Disclosure

All authors have read and approved the final version of the manuscript.

## Conflicts of Interest

The authors declare no conflicts of interest.

## Supporting information


**Supporting Information** Additional supporting information can be found online in the Supporting Information section. Table S1: Data extraction form example. Table S2: Quality assessment of cross‐sectional studies using the Axis‐checklists. Table S3: Quality assessment of prospective and retrospective observational cohort studies using the CASP‐checklists.

## Data Availability

Data sharing is not applicable to this article as no datasets were generated or analyzed during the current study.
