# Supplementary material for: Adapted Screening Methods for Gestational Diabetes in Low‐Resource Settings—A Narrative Review of Observational Studies
Source: J Pregnancy. 2026 Oct 30;2026:6473503. doi: 10.1155/jp/6473503 (PMC13721168; doi:10.1155/jp/6473503)
Supplement: Supplementary file 1 [file JP-2026-6473503-s001.docx]

**Supplementary material**

**Table S1: Data extraction form example**

| **Field** | **Description** |
| --- | --- |
| Study identification | Author, Year, Title |
| Country | Study setting |
| Study Design | Cross-sectional, prospective/retrospective observational |
| Study Period | Period of participant recruitment and data collection |
| Sample Size | Number of participants |
| Screening method and Diagnostic criteria | The GDM screening method that was investigated in the study and the underlying diagnostic criteria, that served as comparison |
| Method of Analysis | Method of statistical analysis used in the studies |
| Sensitivity (%), Specificity (%) | If applicable, percentages of sensitivity and specificity were extracted |
| Outcome | The main outcome and potential applicability was reported |

**Table S2: Quality assessment of cross-sectional studies using the Axis-checklists**

| Questions | Agarwal (2018) | Khambule (2026) | Nwali (2021) | Bhavad-harini (2015) | Putoto (2020) |
| --- | --- | --- | --- | --- | --- |
| 1. Were the aims/objectives of the study clear? | yes | yes | yes | yes | yes |
| 2. Was the study design appropriate for the stated aim(s)? | yes | yes | yes | yes | unclear |
| 3. Was the sample size justified? | no | yes | yes | no | no |
| 4. Was the target/reference population clearly defined? | yes | yes | yes | yes | yes |
| 5. Was the sample frame taken from an appropriate population base so that it closely represented the target/reference population under investigation? | yes | yes | unclear | yes | yes |
| 6. Was the selection process likely to select subjects/participants that were representative of the target/reference population under investigation? | yes | unclear | yes | unclear | yes |
| 7. Were measures undertaken to address and categorise non-responders? | unclear | no | no | unclear | unclear |
| 8. Were the risk factor and outcome variables measured appropriate to the aims of the study? | yes | yes | yes | yes | yes |
| 9. Were the risk factor and outcome variables measured correctly using instruments/measurements that had been trialled, piloted or published previously? | unclear | yes | yes | yes | yes |
| 10. Is it clear what was used to determined statistical significance and/or precision estimates? | yes | yes | yes | yes | yes |
| 11. Were the methods (including statistical methods) sufficiently described to enable them to be repeated? | yes | yes | yes | yes | yes |
| 12. Were the basic data adequately described? | no | yes | no | unclear | unclear |
| 13. Does the response rate raise concerns about non-response bias? | unclear | no | unclear | yes | no |
| 14. If appropriate, was information about non-responders described? | unclear | no | unclear | unclear | yes |
| 15. Were the results internally consistent? | yes | yes | yes | yes | yes |
| 16. Were the results presented for all the analyses described in the methods? | yes | yes | yes | yes | yes |
| 17. Were the authors' discussions and conclusions justified by the results? | yes | yes | yes | yes | yes |
| 18. Were the limitations of the study discussed? | yes | yes | unclear | yes | yes |
| 19. Were there any funding sources or conflicts of interest that may affect the authors’ interpretation of the results? | yes | yes | yes | yes | yes |
| 20. Was ethical approval or consent of participants attained? | yes | yes | yes | yes | yes |
|  | 14/20=70% | 16/20=80% | 15/20=75% | 15/20=75% | 15/20=75% |

Source: Downes MJ, Brennan ML, Williams HC, Dean RS. Development of a critical appraisal tool to assess the quality of cross‑sectional studies (AXIS). BMJ Open. 2016 Dec;6(12):e011458.

**Table S3: Quality assessment of prospective and retrospective observational cohort studies using the CASP-checklists**

| Questions | Olagbuji (2015) | Punnose (2019) | Saravanan (2024) | Dubey (2019) | Soumya (2015) |
| --- | --- | --- | --- | --- | --- |
| 1. Did the study address a clearly focused issue? | yes | yes | yes | yes | yes |
| 2. Was the cohort recruited in an acceptable way? | yes | unclear | yes | yes | unclear |
| 3. Was the exposure accurately measured to minimise bias? | yes | yes | yes | yes | yes |
| 4. Was the outcome accurately measured to minimise bias? | unclear | yes | yes | yes | yes |
| 5. (a) Have the authors identified all important confounding factors? | yes | yes | no | unclear | no |
| b) Have they taken account of the confounding factors in the design and/or analysis? | yes | yes | no | unclear | no |
| 6. a) Was the follow up of subjects complete enough? | yes | yes | yes | yes | yes |
| b) Was the follow up of subjects long enough? | yes | yes | yes | yes | yes |
| 7. What are the results of this study? | yes | yes | yes | yes | yes |
| 8. How precise are the results? | yes | yes | yes | yes | yes |
| 9. Do you believe the results? | yes | yes | yes | yes | yes |
| 10. Can the results be applied to the local population? | yes | unclear | yes | yes | yes |
| 11. Do the results of this study fit with other available evidence? | yes | yes | yes | yes | yes |
| 12. What are the implications of this study for practice? | unclear | unclear | yes | unclear | yes |
|  | 10/12=83% | 09/12=75% | 10/12=83% | 09/12=75% | 09/12=75% |

Source: Critical Appraisal Skills Programme. CASP Cohort Study Checklist [Internet]. Oxford: CASP; 2025 [zitiert am 29.06.2025]. Verfügbar unter: https://casp-uk.net/casp-tools-checklists/
